# Energy balance-related parenting and child-care practices: The importance of meso-system consistency

**DOI:** 10.1371/journal.pone.0203689

**Published:** 2018-09-07

**Authors:** Jessica S. Gubbels, Kelly Stessen, Ilona van de Kolk, Nanne K. de Vries, Carel Thijs, Stef P. J. Kremers

**Affiliations:** 1 Department of Health Promotion, Faculty of Health, Medicine and Life Sciences, Maastricht University, Maastricht, the Netherlands; 2 NUTRIM School of Nutrition and Translational Research in Metabolism, Maastricht University, Maastricht, the Netherlands; 3 CAPHRI Care and Public Health Research Institute, Maastricht University, Maastricht, the Netherlands; 4 Department of Epidemiology, Faculty of Health, Medicine and Life Sciences, Maastricht University, Maastricht, the Netherlands; TNO, NETHERLANDS

## Abstract

**Background:**

Our knowledge of the role of parental and child-care staff behavior in the development and prevention of obesity is rapidly increasing. Potential interaction between both settings in so-called meso-systems, as hypothesized by the ecological systems perspective, is however often ignored. Specifically, inconsistency between home and child-care is hypothesized to have negative effects on child outcomes.

**Methods:**

Participants were recruited through 23 child-care centers in the Netherlands. Data regarding 161 child-parent-child-care staff triads were available. Parenting and child care practices were assessed using validated questionnaires for parents (Child Feeding Practices Questionnaire, Preschooler Physical Activity Parenting Practices instrument) and child-care staff (Child-care Food and Activity Practices Questionnaire), using similar items in both settings. Absolute difference scores between parents and child-care staff were calculated for each triad as a measure of meso-system consistency. Child outcomes were physical activity (as assessed by accelerometry), dietary intake (from the parental questionnaire), and measured BMI z-scores. Paired t-tests were used to examine consistency between practices in both settings. Linear regression analyses were used to explore the association of parenting practices, child-care practices and difference scores on the one hand, and child outcomes on the other.

**Results:**

Significant differences between settings were found for almost all practices, and in most cases child-care staff scores more favorable on the practices than parents. Inconsistencies were mostly associated with unhealthy dietary intake and lower physical activity levels, but not with BMI.

**Conclusion:**

The current study showed that inconsistencies in parenting and child-care practices exist, and that these inconsistencies seem to be associated with unhealthy behavior in children. The results underline the importance of studying meso-system influences on behavior in general, and children’s energy balance-related behavior specifically.

## Introduction

Various studies seem to indicate an increased overweight risk in children attending child-care (e.g. [1–4]), although this association seems to be dependent on a number of factors, including age at initiation and type of care [5]. Nonetheless, child-care and early education facilities (including pre-school, kindergarten, day-care) could play an important role in childhood overweight prevention [6]. Not only parents [7, 8], but also child-care workers’ practices have an important influence on children’s dietary intake and physical activity [9]. Specifically, responsive strategies that enable child control over eating seem benificial [9, 10]. Encouragement and modeling of healthy behaviors by child-care staff also seems very promising [9, 11–13]. In line with this, positive supporting approaches by parents, stimulating intake of healthy foods and being physically active and being a positive role model, consistently show positive effects [7, 8]. On the other hand, emotional feeding practices, in which food is used by parents to comfort a child, are consistently associated with undesirable outcomes [7].

While our knowledge of the role of parental and child-care staff behavior in the development and prevention of obesity is rapidly increasing, studies into determinants of children’s behavior and weight status have almost exclusively focused on examination of the influence of either child-care *or* home influences [14]. However, the ecological systems perspective of environmental influences on human behavior [15, 16] argues that settings, such as the home and child-care setting, interact with each other in influencing children’s behavior and weight status [17, 18]. The influence of the child-care environment thus depends on what happens at home, as well as the other way around [14]. Within the ecological systems perspective, interaction between settings, or so-called ‘micro-systems’, has been defined as the ‘meso-system’ [19]. Although quantitative studies examining the home-child-care meso-system are lacking [14], qualitative research provides strong indications that interaction between settings exists. Several qualitative studies among child-care workers highlighted the importance of support of and communication with parents for promoting sufficient physical activity and healthy dietary intake at child-care [20–23]. Parents were explicitly mentioned by child-care staff as an obstacle to physical activity, as parents restricted children’s activities at the child-care centers due to a variety of reasons (e.g. parents limiting time children were allowed to spend outdoors at child-care, letting children wear clothes unsuitable for physical activity, communicating that the child is not able yet to perform certain activities, prioritizing academic performance, safety concerns [22–27]). Similarly, parents were also commonly mentioned as a barrier for promoting healthy nutrition in child-care [28, 29], and child-care staff even used certain feeding practices despite knowing these would have adverse effects, out of fear for parents’ reactions if a child would not have eaten enough at child-care [30]. In addition to parents influencing behavior in the child-care setting, child-care workers also indicated interaction the other way around: they advised parents regarding children’s physical activity and nutrition and were aware that they could be a role model for parents [23, 25, 31], but also felt uncomfortable addressing weight-related issues with parents [31, 32]. Parents recognized that child-care practices influenced the home situation and child’s behavior at home, both positively and negatively [33, 34]. Parents also actively sought parenting advice from child-care workers [21]. Inconsistency between the child-care setting and home was perceived as hampering physical activity at child-care [20]. Also for interventions, Nader and colleagues have argued the importance of aligning obesity prevention messages from different sources to create synergistic effects [35].

The notion that inconsistency might result in negative effects on child outcomes is not new. Although quantitative studies examining home-child-care meso-system inconsistency are lacking [14], previous research has examined consistency on a more proximal level: within the home micro-setting. Some studies have shown that inconsistencies between parents with regard to parenting practices are associated with undesirable outcomes [36, 37]. If fathers and mothers showed large differences between their practices, their practices less often had the intended desirable effects on snack intake [37]. Inconsistency of parenting between fathers and mothers was further associated with higher child BMI [36]. Zooming in even further, we can see that also within one caregiver consistency is important: the parenting dimension Structure, reflecting consistency over time, has been shown to be associated with child outcomes [38].

The current study examines the role of inconsistency between parents’ and child-care workers’ practices, based on the ecological perspective. In line with the studies described above, we hypothesize that inconsistency is associated with undesirable child outcomes.

## Materials and methods

### Respondents and procedure

Formal center-based child-care in the Netherlands is typically organized in centers that house one or more groups of around 15 children during the day. Opening hours vary around 10–12 hours, from 7-8am to 6-7pm. Depending on the age of the children, two to three staff members supervise a group. Parents can get funding for child-care from the Dutch government, depending on parents’ income and working hours.

A total of 174 child-care centers in the southern provinces of the Netherlands (Noord-Brabant and Limburg) were approached to participate in the current study. Twenty-three child-care centers (13.2%) agreed to participate in the study. All parents of the children aged 1 to 4 years old from these child-care centers were invited to participate either by letter or email, as well as face-to-face on-site recruitment at drop-off and pick-up times. In total, parents of 218 children agreed to participate. All parents of participating children provided written informed consent. In addition, participating child-care staff also provided written informed consent. Eight children were excluded because of severe food intolerances or allergies, not being able to walk independently or being indicated as too young for the study by the parents, resulting in a sample of 210 children. The data collection period ranged from November 2014 to January 2016. The study was approved by the Maastricht University Medical Centre+ medical ethics committee.

For each child-parent-child-care staff member triad, several measures were completed: Parents and child-care staff filled out a questionnaire, children were measured and weighed at the child-care center, and children wore accelerometers to assess physical activity. For 161 triads (76.7%) the parenting practices questionnaire and child-care staff practices questionnaire were available. These triads were included in the analyses. Reasons for missing data were for instance absence of the child due to illness on the measurement day. The measures are described in more detail below.

### Measures

#### Parenting practices, child-care practices, and inconsistencies

Parents filled out a questionnaire regarding their parenting practices. Parenting practices were assessed using the validated Child Feeding Practices Questionnaire (CFPQ, [39]) for diet-related practices and Preschooler Physical Activity Parenting Practices (PPAPP) instrument [40] for physical activity-related practices. Child-care workers’ diet- and physical activity-related practices were assessed using the Child-care Food and Activity Practices Questionnaire (CFAPQ, [41]). One child-care worker per group filled out the questionnaire for the whole group, not per individual child. Diet-related items were answered using a five-point Likert scale ranging from ‘never’ to ‘always’ for questions, and from ‘disagree’ to ‘agree’ for the statements. All activity-related items were answered on a five-point Likert-scale ranging from ‘never’ to ‘always’. The CFAPQ was designed by converting items of the CFPQ [39] and PPAPP [40] to items suitable for the child-care setting. Using the same items in both settings allows for comparison between both settings, ruling out the possibility that measurement differences cause spurious inconsistencies between both settings, instead of measuring actual inconsistencies [41].

Nine out of 49 items of the CFPQ [39] and 9 items of the 32 item PPAPP questionnaire [40] were dropped because they were not included in the 63 items of the CFAPQ (as they were not applicable to the child-care setting [41]), and could thus not be compared between settings. 11 items of the CFAPQ were dropped because a combination of the items being considered less applicable for the Dutch setting and space limitations in the questionnaires. For example, the items assessing videogame use and long periods of TV watching were dropped, as this is not common in Dutch child-care. This resulted in 52 items being assessed in both the parental sample and the child-care staff sample. The scales of the CFPQ and PPAPP were applied to both the parenting practices items (as assessed by the CFPQ and PPAPP) and the child-care practices items (as assessed by the CFAPQ).

To ensure comparability between and scale reliability in both settings, the same scales had to be applied to both the child-care items and the parental items. The following procedure was used. Reliability for the child-care instrument was examined by calculating Cronbach’s alpha values, applying the CFPQ and PPAPP scales. Items were removed until all scales reached acceptable Cronbach’s alphas (>.50; [42]). These adjusted scales were then applied to the parental items, subsequently calculating Cronbach’s alpha’s and removing items until all scales reached acceptable Cronbach’s alpha’s in the parental sample as well. Next, these adjusted scales were again applied to the child-care items to check whether these second time adjusted scales were still reliable in the child-care sample. This procedure resulted in several single items, in addition to the scales. The items of each reliable scale were added and divided by the number of items to calculate the average score for each practice. The final scales and their reliability, as well as the single items, are presented in Table 1.

**Table 1:** Descriptive and scale information of and differences between parenting and child-care staff practices^a^ (N = 161).

Category	Practice	Number of items	Parental sample		Child-care staff sample		p-value difference^b^
			*Cronbach ‘s alpha*	*Mean (sd)*	*Cronbach ‘s alpha*	*Mean (sd)*	
Diet-related	Restriction	5	.56	3.31 (.65)	.63	3.71 (.69)	<.001
	Modeling	4	.73	4.22 (.59)	.63	4.51 (.52)	<.001
	Encourage balance and variety	3	.57	4.31 (.59)	.63	4.44 (.55)	.040
	*Single item*: How often do you encourage your child/the children to eat healthy foods before unhealthy ones?	1	-	3.93 (1.09)	-	4.37 (1.04)	.001
	Environment	2	.52	3.70 (.86)	.59	4.58 (.60)	<.001
	*Single item*: I keep / There is a lot of snack food present in my house / the child-care center. (mirrored)	1	-	3.53 (1.12)	-	4.59 (.92)	<.001
	*Single item*: A variety of healthy foods are available to my child / the children at each meal served at home / the child-care center.	1	-	3.45 (1.08)	-	4.44 (.90)	<.001
	Teaching about nutrition	2	.67	3.29 (1.15)	.64	3.50 (.93)	.070
	*Single item*: I tell my child / the children what to eat and what not to eat without explanation. (mirrored)	1	-	3.86 (.97)	-	4.38 (.97)	<.001
	Pressure to eat	4	.70	3.05 (.85)	.70	3.26 (.79)	.025
	Child control	5	.51	2.45 (.52)	.57	2.66 (.56)	<.001
	Emotion regulation						
	*Single item*: How often when this / a child gets fussy, is giving him/her something to eat the first thing you do?	1	-	1.76 (.73)	-	1.37 (.58)	<.001
	*Single item*: How often do you give this / a child something to eat or drink if s/he is upset, even if you think s/he is not hungry?	1	-	1.39 (.64)	-	1.27 (.53)	.054
	*Single item*: How often do you give this / a child something to eat or drink if s/he is bored, even if you think s/he is not hungry?	1	-	1.28 (.53)	-	1.11 (.37)	.001
Activity-related	Parental/child-care worker engagement and structure	12	.83	3.43 (.45)	.89	3.80 (.47)	<.001
	Psychological control	3	.59	2.42 (.68)	.52	2.30 (.68)	.101
	*Single item*: How often do you not let your child / children play actively for fear of him/her / them getting dirty?	1	-	1.54 (.71)	-	1.27 (.74)	.001
	*Single item*: How often do you tell your child s/he is / children they are not good enough yet at sports or active games?	1	-	1.38 (.65)	-	1.05 (.22)	<.001
	Promote inactivity	2	.56	2.13 (.82)	.54	1.84 (.69)	<.001
	*Single item*: How often do you have outdoor toys available for your child / the children?	1	-	4.43 (.77)	-	4.58 (.52)	.035

^a^ Parenting and child-care practices measure by CFPQ, PPAPP and CFAPQ (Scale 1–5).

^b^ P-value from t-test comparing parenting practice and child-care practice.

Home-child-care inconsistencies were operationalised by the absolute difference score between the parenting practice and child-care practice score on each scale and single item.

#### Children’s physical activity, dietary intake, BMI and background characteristics

Children’s physical activity level was objectively measured using an accelerometer (Actigraph GT3X+, 30 Hz, Actigraph Pensacola, FL), applying a validated wearing protocol [43]. The epoch was set to 15 seconds. Children wore the accelerometer for 7 consecutive days on the right hip during waking hours, except during swimming, showering and other activities involving water. To be included in the analyses, minimal wear time per day was 360 minutes. Based on a review of cut points for toddlers [44], cut points by Pate and colleagues [45] were applied to extract the time spent sedentary and in moderate to vigorous physical activity (MVPA). Time spent in these categories was divided by total wearing time to calculate percentage of wearing time spent sedentary and in MVPA. In addition, average counts per minute based on vector magnitude was extracted.

Overall dietary intake (including both intake at home and elsewhere) was assessed using a food frequency questionnaire in the parental questionnaire. Parents were asked how often their child consumed biscuits and cake, sweets, savory snacks, water (including unsweetened tea) and sweet beverages (including processed fruit juices; excluding fresh fruit juice). Answering options were ‘never or less than once a week’, ‘1–3 times a week’, ‘4–6 times a week’, ‘once a day’, ‘twice a day’, and ‘3 or more times a day’. Intake was recoded into weekly intake frequency using the midpoint value of each category comprising a range (e.g. 2 times a week for the category ‘1–3 times a week’).

Children’s height and weight were measured at the child-care center by trained research assistants. Height and weight were used to calculate body mass index (BMI), which was converted to BMI z-scores, reflecting the number of standard deviations the child differed from the age- and gender-specific mean of the national reference population [46].

In addition, several background characteristics were assessed through the parental questionnaire: which parent filled out the questionnaire (mother, father or together), gender and age (in months) of the child, parental age (in years), country of birth (the Netherlands vs. other) and BMI (in kg/m^2^, calculated from reported weight and height) of both parents, child-care use of the child (number of half-days per week), and educational level of the parents). Child-care use was converted into whole days. Educational level was converted into low (elementary school, lower secondary education, lower vocational education), medium (medium vocational education, higher secondary education and college prep) and high (higher vocational education, university); and then recoded into dummy variables for low and high compared to medium.

Finally, gender, age (in years) and BMI (in kg/m^2^, calculated from reported weight and height) of the child-care staff were assessed through the questionnaire for child-care staff.

### Statistical analyses

All analyses were conducted using SPSS 24.0. *p*-values <.05 were considered statistically significant. Descriptive statistics were used to examine all variables included in the study. Paired T-tests were used to compare parenting practices to child-care staff practices, for each individual practice (scales and single items).

Multiple linear regression analyses were conducted to examine the associations between parenting practices on the one hand, and children’s overall dietary intake (biscuits and cake, sweets, savory snacks, water and sweet beverages), total physical activity (average counts/minute, percentage sedentary, percentage MVPA) and BMI z-score, adjusted for children’s background variables as described above (questionnaire completer; age, gender and child-care usage of the child; age, country of birth, educational level and BMI of both parents; age and BMI of the child-care staff member). This procedure was then repeated for staff practices, creating a new set of regression analyses. In a final set of regression analyses, the absolute difference scores between each parenting and child-care practice were added to the model with both parenting and child-care staff practices, as well as children’s background characteristics. Considering the exploratory nature of the meso-system research question, the number of variables in the final models (including the difference scores) and the consequential risk of suppression, trends toward statistical significance (p <.10) were also assessed.

## Results

Background characteristics of the children, parents and child-care staff are presented in Table 2. There were slightly more girls (52.5%) than boys. Mean age of the children was 2 years and 10 months, and they used child-care for an average of 2 days a week. Questionnaires were mostly filled out by mothers (82.4%), and most parents had a high educational level (66.0 and 62.3% for questionnaire completers and their partners, respectively). Mean parental BMI (24.6 and 24.7, respectively) was somewhat higher than mean child-care staff BMI (22.6). Most child-care staff was female (94.6%).

**Table 2:** Descriptives of background characteristics and outcome variables (N = 161).

			N (%)^a^	Mean (sd)
**Child**	Gender	*Boy*	76 (47.5%)	
		*Girl*	84 (52.5%)	
	Age (months)			34.4 (9.1)
	BMI z-score			.35 (.95)
	Child-care use			2.0 (0.8)
	Dietary intake (times per week)	*Biscuits and cake*		4.6 (2.9)
		*Sweets*		4.0 (4.2)
		*Savory snacks*		2.4 (2.9)
		*Water*		10.4 (7.8)
		*Sweet drinks*		11.0 (6.6)
	Physical activity	*Average counts/minute*		1130.3 (223.7)
		*Percentage sedentary*		81.8 (4.5)
		*Percentage MVPA*		8.7 (3.1)
**Questionnaire completer**		*Mother*	131 (82.4%)	
		*Father*	18 (11.3%)	
		*Together*	10 (6.2%)	
	Age (years)			34.7 (4.2)
	County of birth	*The Netherlands*	153 (95.6%)	
		*Other*	7 (4.4%)	
	Educational level	*Low*	8 (5.0%)	
		*Medium*	46 (28.9%)	
		*High*	105 (66.0%)	
	BMI			24.6 (3.8)
**Partner**	Age (years)			36.8 (4.4)
	County of birth	*The Netherlands*	149 (95.5%)	
		*Other*	7 (4.5%)	
	Educational level	*Low*	12 (7.8%)	
		*Medium*	46 (29.9%)	
		*High*	96 (62.3%)	
	BMI			24.7 (2.8)
**Child-care staff**	Gender	*Female*	35 (94.6%)	
		*Male*	2 (5.4%)	
	Age (years)			39.3 (11.2)
	BMI			22.6 (2.6)

Notes: BMI: body mass index, MVPA: moderate to vigorous activity, sd: standard deviation

^a^ N’s deviate from total sample size due to missing values. Valid percentages are presented.

Children were somewhat heavier than the reference population [46], with an average BMI z-score of .35 (see Table 2). They drank sweet drinks more often (11.0 times a week) than water (10.4 times). Children were sedentary for the larger part of the time (81.8%), and accumulated 8.7% MVPA. The means of parents and child-care staff on the practices can be found in Table 1. Parents scored high (>4.0 on a scale 1–5) on diet-related modeling and encouraging balance and variety, as well as having outdoor toys available for the children. Child-care staff scored high on these practices as well, and additionally on encouraging children to eat healthy foods before unhealthy ones, keeping few snack foods around, and having a variety of healthy foods available. Parents and child-care staff both scored low (<2.0) on the diet-related emotion regulation and activity-related psychological control single items. Additionally, child-care staff, but not parents, scored low on promoting inactivity.

### Associations of parenting and child-care practices with child outcomes

Parents having healthy foods available was significantly associated with children eating less biscuits and cake (standardized regression coefficient β = -.29, p = .04). Parental modeling of healthy food intake was associated with lower savory snack intake of the children (β = -.29, p = .03), but with higher child BMI (β = .32, p = .04). Parental emotion regulation by giving a child food if the child was fussy (β = .37, p = <.01) or bored (β = .23, p = .04) was associated with higher savory snack intake. Parental emotion regulation if a child was upset was associated with higher sweet drink intake (β = .33, p = .01). Parents who encouraged balance and variety had children who ate more savory snacks (β = .29, p = .03).

As regards physical activity parenting practices, parents having outdoor toys available at home was associated with higher overall activity levels (CPM, β = .33, p <.001), more MVPA (β = .27, p = .01), and less sedentary time (β = -.24, p = .01). Parents promoting inactivity was associated with more MVPA (β = .28, p = .04).

With regard to child-care workers, encouraging children to eat healthy foods before unhealthy ones was associated with lower sweets intake (β = -.31, p = .04). Staff modeling of healthy intake was associated with higher water intake (β = .40, p = .02). Use of food to calm a fussy child down was associated with higher sweet drink intake (β = .42, p = .02). Child-care workers teaching about nutrition was associated with higher biscuit and cake (β = .34, p = .04), sweets (β = .42, p <.01) and savory snack (β = .32, p = .04) intake. Child-care workers giving children much control over their intake was associated with lower child BMI (β = -.37, p = .04).

Child-care workers promoting inactivity was associated with lower overall activity level (β = -.28, p = .03), and more sedentary time (β = .39, p <.01). Child-care staff limiting children’s activity out of fear of them getting dirty was associated with more MVPA (β = .32, p = .01).

### Consistency of parenting and child-care practices

For the majority of the practices (16 out of 20), there was a significant difference (p <.05) between parents and child-care staff, with three out of the four remaining practices showing a marginal significant difference (p <.10; see Table 1). For all activity-related practices (5 out of 5) and most diet-related practices (13 out of 15), child-care staff scored more favorably on the practices scales than parents. Exceptions were child-care staff scoring higher on restriction (3.71 vs. 3.31 for the parents) and pressure to eat (3.26 vs. 3.05 for the parents).

### Associations of consistency between parents and child-care staff with child outcomes

Associations of inconsistencies between parents and child-care staff were examined while adjusting for main effects of parents’ and staff practices. The associations are presented in Tables 3 and 4. Inconsistencies with regard to restriction were associated with higher sweet beverages intake (β = .26, p = .02) and marginally associated with higher sweets intake (β = .21, p = .08). Inconsistent emotion regulation if the child was bored was associated with lower water intake (β = -.57, p = .046). Inconsistencies in encouraging balance and variety were associated with higher biscuit and cake intake (β = .52, p <.01). Inconsistencies in pressure to eat were associated with higher sweet beverages intake (β = .29, p = .04). Inconsistencies in modeling of healthy intake were associated with higher savory snack intake (β = .40, p = .03), but lower biscuit and cake intake (β = -.38, p = .03). Inconsistencies in having few snacks around were associated with lower sweet beverages intake (β = -.46, p = .04).

**Table 3:** Association of difference scores between parents and child-care staff practices, with child dietary intake.

	*Standardized regression coefficient (β) for absolute difference score between practices*				
	Biscuits and cake	Sweets	Savory snacks	Water	Sweet beverages
Restriction	.00	**.21**^Ɨ^	-.01	-.09	.26*
Modeling	-.38*	.12	.40*	-.12	-.04
Encourage balance and variety	.52**	-.05	-.10	-.06	.06
Encourage healthy food before unhealthy	.04	-.05	.22	-.20	.27
Environment	.20	.21	-.14	-.05	.05
A lot of snack food present (*m*)	.14	.04	.20	.10	-.46*
Variety of healthy foods available	.21	.**41**^Ɨ^	-.03	.16	.30
Teaching about nutrition	-.21	-.10	-.10	.00	-.02
Tell child what to eat without explanation (*m*)	.13	-.17	-.17	-.05	-.03
Pressure to eat	.05	.04	.05	-.20	.29*
Child control	-.09	-.17	.08	.20	.00
Giving something to eat if child is fussy	-.12	-.05	.05	.10	-.02
Giving something to eat if child is upset	.23	.29	-.06	.11	-.19
Giving something to eat if child is bored	.27	.22	.05	-.57*	.44

^Ɨ^ p <.10,

* p <.05,

** p <.01

Notes: All models are adjusted for the main effects of parenting and child-care staff practices, as well as the following covariates: questionnaire completer; age, gender and child-care usage of the child; age, country of birth, educational level and BMI of both parents; age and BMI of the child-care staff member. (Marginally) significant associations are presented **bold**. Abbreviation: m: item mirrored.

**Table 4:** Association of parenting practices, child-care practice and difference scores between parents and child-care staff, with child physical activity.

	*Standardized regression coefficient (β) for absolute difference score between practices*		
	CPM	Sedentary time	MVPA
Engagement and structure	.06	-.04	-.01
Psychological control	-.11	.09	-.10
Fear of child getting dirty	**-.34**^Ɨ^	.30	.24
Child not good enough at PA	-.09	-.07	.03
Promote inactivity	-**.18**^Ɨ^	.16	**-.26**^Ɨ^
*O*utdoor toys available	.08	-.01	.01

^Ɨ^ p <.10

Notes: All models are adjusted for the main effects of parenting and child-care staff practices, as well as the following covariates: questionnaire completer; age, gender and child-care usage of the child; age, country of birth, educational level and BMI of both parents; age and BMI of the child-care staff member. (Marginally) significant associations are presented **bold**.

As regards physical activity, inconsistencies between parents and child-care staff with regard to promoting inactivity showed a trend for being associated with less MVPA (β = -.26, p = .08), as well as for a lower CPM (β = -.18, p = .099). Inconsistencies in letting children not being active out of fear of them getting dirty, was marginally significantly associated with lower average activity levels (CPM, β = -.34, p = .06).

## Discussion

The current study explored the association between inconsistency of practices in the home and child-care setting on the one hand, and children’s diet, physical activity and BMI on the other. The research question is in line with the hypothesized existence of meso-systems (interactions between two or more micro-systems), as proposed by the ecological systems perspective [17]. This study showed the existence of inconsistencies in energy balance-related practices between child-care and home, with the large majority of examined practices showing significant differences between both settings. Overall, child-care staff scored more favorably than parents, with higher scores on desirable practices and lower scores on undesirable practices. This seems logical, as working in child-care requires a pedagogical education, while most parents have not had any preparation for parenthood. Moreover, child-care workers are guided and constrained by center and governmental policies and regulations, and have certain standardized play facilities (e.g. outdoor play space) and foods available at child-care, which does not apply to parents.

As for the effect of inconsistency on the child, most associations pointed in the direction that inconsistencies were associated with unhealthy child outcomes. However, there were a few associations pointing in the opposite direction, and a large number of non-significant associations. Hence, cautious interpretation is needed, also in light of the limitations of the current study. Nonetheless, the findings mostly seem to confirm the hypothesis that meso-system inconsistencies have negative effects, in line with qualitative studies (see e.g. [14]). In fact, the data showed several practices from individual settings which did not contribute to child outcomes, while the discrepancy between the settings did explain child outcomes. For example, for both pressure to eat and restriction, there were no main effects of the use of the practice by parents or child-care staff. However, for both practices, larger difference scores between both caregivers (parents and staff) were significantly associated with higher sweet drink intake by the child, even when controlled for the main effects of each setting. This underlines the added value of examining meso-systems in addition to micro systems, applying an ecological systems view. As for the child, one can imagine the confusion that arises in this situation: one day your feelings of hunger and satiety are respected, while the next day you are pressured to clear your plate or continue eating, disregarding those same feelings and consequently subverting the innate ability to self-regulate energy intake (see e.g. [47]).

Also general child development literature points in the direction that inconsistencies in parents’ and child-care staff’s general child-rearing practices affect children’s wellbeing negatively [48]. This underlines basic assumptions of the ecological perspective: the stronger the link between two micro-systems, the better the child’s outcomes [49]. The problem is that while the child participates fully in these two micro-systems with different demands, parents and child-care staff do not have a direct link with the other micro-system [49]. This link can be created through communication: Communication between home and child-care improves child-care quality [50], and creates synergy and consistency, supporting optimal child development [51]. Parents and supervisors can, for instance, make agreements on the foods that should be offered to the child. However, there are numerous barriers for communication between child-care providers and parents, including parents being too busy and child-care providers being afraid to offend parents [52]. Clear, written policies can facilitate communication [52], giving child-care workers a tool they can refer to and fall back on. However, such policies are often lacking for physical activity and sedentary time [53], and are often not comprehensive for nutrition [28]. In addition, it is important to realize that policies are not a magic bullet: they have no use if they are not acted upon by staff. Implementation of state level nutrition policies in the US, for example, led to only modest improvements in child-care nutrition practices, showing that policy implementation needs to be coupled with center level support to ensure centers and staff comply with these policies [54]. Policy changes should thus be embedded in comprehensive multilevel programs. Parental involvement in such programs is crucial: A recent review of obesity prevention interventions in early care and education settings showed that child-care interventions which include parental engagement are most effective [55]. The same has been concluded for interventions in schools [56]; Okely and Hammersley recently suggested that school-home partnerships might be ‘the missing piece’ in our obesity prevention efforts [57]. Our study extends this conclusion, by indicating that parents should not only be included in programs, but that such a parental component should also be consistent with the child-care (or school) component, in order for parents and child-care workers to convey the same messages to the child and avoid inconsistencies. Assuming our cautious conclusion that inconsistency can have negative effects on the child is correct, this could even imply that single-setting interventions can potentially be harmful if they create or increase inconsistencies between settings. Perhaps focussing on small instead of large changes in child-care interventions is crucial, avoiding to create large discrepancies with the home environment. Small steps leave room for transfer of intervention effects from child-care to the home setting, graduately moving *both* settings in the desired direction. Opposing these hypotheses, however, some authors propose that for children’s cognitive and socioemotional development, high child-care quality is particularly important for children from low-income families [58], and that high-quality child-care can diminish the adverse effects of a disadvantaged home [18, 51]. In this respect, it would be interesting to specifically look at the direction of discrepancies: in the current study childcare staff mostly scored more favorable on practices than parents; it is not clear whether effects of inconsistencies are the same when the home setting is more favorable than the child-care setting. In addition, the intensity of child-care use might play a role. Children in the current study used child-care for an average of 2 days a week. The majority of the week they were thus cared for elsewhere, by either their parents or another formal or informal caregiver. Meso-system (in)consistencies might become increasingly important with increasing child-care use. Thus, the central question seems to be under which circumstances two micro-systems can reach meso-system synergy. More research will be needed to examine these assumptions and hypotheses in the context of obesity [14].

A growing number of studies recognize the multivariate and multilevel structure of determinants of behavior [14], or as Hawe and colleagues [59] put it: ‘It is hard these days to find a health promotion program that does not claim to take an ecological approach’. Various studies examine extensive multilevel lists of determinants of childhood obesity (e.g. [60–62], but relationships *between* determinants are often ignored [59], disregarding the assumption of interaction between behavioral determinants of the ecological perspective [14, 63]. We therefore call for examination of interaction between determinants and settings, potentially revealing meso-systems contributing to childhood obesity. Other meso-systems of potential interest include the home-school meso-system, but also the meso-system created by co-parenting of divorced parents. Qualitative research by Khandpur and colleagues [64] showed that 40% of divorced parents are not in line with each other with regard to feeding practices, often reflected in one parent undermining the other’s efforts or overcompensation between parents. In line with current findings, such inconsistencies were found to result in unfavorable child outcomes, including child tantrums, manipulation and refusal to eat [64].

There is another important factor to consider when examining meso-systems influences. De Schipper and colleagues reported that especially for children with a more difficult temperament, several parallel care arrangements can interfere with the process of adapting to the child-care setting [65]. This indicates a three-way interaction between home, child-care and child temperament. Likewise, the number of days a child attends childcare might also influence the ability to cope with meso-system inconsistencies. The existence of such interactions could possibly explain some of mixed findings in the current study, as two of the associations of inconsistency actually showed positive effects (i.e. inconsistency in modeling of healthy intake was associated with lower biscuit and cake intake and inconsistency in having snacks around was associated with lower sweet drink intake). Depending on personal characteristics, one child might be better able to cope with meso-system inconsistencies than another. This assumption extends previous research showing that children respond differently to micro-system influences as well, depending on their temperament and other characteristics (see e.g. [66, 67]).

The current study has several strengths and limitations that need to be taken into account. A strength of the study is its design, which provided data regarding 161 child-parent-child-care staff triads. The same items for assessing diet- and activity-related practices were used in both settings, from questionnaires validated in both settings [39–41]. In addition, child physical activity and BMI were assessed objectively by accelerometers and trained research assistants, respectively. However, overall dietary intake (including intake outside of the home setting) was reported by parents, potentially resulting in bias. Additionally, we only looked at overall physical activity and dietary intake. It would be interesting to look at the associations of (in)consistency on behaviors in each setting separately. Furthermore, the sample size was limited, hampering correction for the multi-level structure of the data. Recruitment of child-care centers was challenging, as the Dutch child-care system was in a transitional phase during the research period, resulting in circumstances hampering recruitment (e.g. center bankruptcies). Furthermore, child-care practices of only one staff-member per group were taken into account in the current study. Additionally, highly educated parents were overrepresented. Furthermore, the combination of various outcomes and various practices resulted in a large number of associations examined, which increases the odds of false positive associations (type I errors). In addition, the data were cross-sectional, not allowing for causal inferences. Reverse causation could explain the contra-intuitive main effects of some practices (e.g. the positive association between MVPA and parents and staff promoting inactivity). In addition, while we assumed that inconsistency between settings caused undesirable child outcomes, one could also reason that parents and child-care staff might respond differently to undesirable child behavior (i.e. inactivity or unhealthy dietary intake), switching cause and consequence. However, child-care practices were assessed on group level, making it unlikely that child-care staff adapted their practices in response to an individual child’s behavior. Longitudinal studies and intervention studies (aimed at promoting healthier practices while reducing inconsistencies and improving communication) with large sample sizes will be needed to further examine causality of the associations found in the current study. Furthermore, additional child outcomes should be included in future studies. Fruit and vegetable consumption were not included in the current study, for instance.

## Conclusions

In conclusion, the current study showed that inconsistencies in parenting and child-care practices exist, and that these inconsistencies seem to be associated with lower diet quality and lower activity levels in children. While qualitative studies provide us with ample indications of meso-system interactions, quantitative research is still lacking. We call for increased recognition of between-settings interaction in future research.

## Supporting information

S1 File CFPQ, PPAPP and CFAPQ items for parents and child-care staff in Dutch and English. (DOC)

S2 File Data used for the manuscript. (SAV)

## References

[1] Gubbels JS, Kremers SP, Stafleu A, Dagnelie PC, de Vries NK, van Buuren S, et al Child-care use and the association with body mass index and overweight in children from 7 months to 2 years of age. Int J Obes (Lond). 2010;34(10):1480–6. 20498654 10.1038/ijo.2010.100

[2] Benjamin SE, Rifas-Shiman SL, Taveras EM, Haines J, Finkelstein J, Kleinman K, et al Early child care and adiposity at ages 1 and 3 years. Pediatrics. 2009;124(2):555–62. 10.1542/peds.2008-2857 19651579 PMC3049895

[3] Maher EJ, Li G, Carter L, Johnson DB. Preschool child care participation and obesity at the start of kindergarten. Pediatrics. 2008;122(2):322–30. 10.1542/peds.2007-2233 18676550

[4] Geoffroy MC, Power C, Touchette E, Dubois L, Boivin M, Seguin JR, et al Childcare and overweight or obesity over 10 years of follow-up. J Pediatr. 2013;162(4):753–8 e1. 10.1016/j.jpeds.2012.09.026 23140878

[5] Alberdi G, McNamara AE, Lindsay KL, Scully HA, Horan MH, Gibney ER, et al The association between childcare and risk of childhood overweight and obesity in children aged 5 years and under: a systematic review. Eur J Pediatris. 2016;175(10):1277–94. 10.1007/s00431-016-2768-9 27631590

[6] Story M, Kaphingst KM, French S. The role of child care settings in obesity prevention. Future Child. 2006;16(1):143–68. 16532662 10.1353/foc.2006.0010

[7] Larsen JK, Hermans RCJ, Sleddens EFC, Engels RC, Fisher JO, Kremers SPJ. How parental dietary behavior and food parenting practices affect children’s dietary behavior. Interacting sources of influence?. Appetite. 2015;89:246–57. 10.1016/j.appet.2015.02.012 25681294

[8] Sleddens EF, Kremers SP, Hughes SO, Cross MB, Thijs C, De Vries NK, et al Physical activity parenting: a systematic review of questionnaires and their associations with child activity levels. Obes Rev. 2012;13(11):1015–33. 10.1111/j.1467-789X.2012.01018.x 22845791

[9] Ward S, Belanger M, Donovan D, Carrier N. Systematic review of the relationship between childcare educators’ practices and preschoolers’ physical activity and eating behaviours. Obes Rev. 2015;16(12):1055–70. 10.1111/obr.12315 26345462

[10] Blaine RE, Davison KK, Hesketh K, Taveras EM, Gillman MW, Benjamin Neelon SE. Child Care Provider Adherence to Infant and Toddler Feeding Recommendations: Findings from the Baby Nutrition and Physical Activity Self-Assessment for Child Care (Baby NAP SACC) Study. Childhood obesity. 2015;11(3):304–13. 10.1089/chi.2014.0099 25918873 PMC4485887

[11] Gubbels JS, Gerards SMPL, Kremers SPJ. Use of food practices by childcare staff and the association with dietary intake of children at childcare. Nutrients. 2015;7:2161–75. 10.3390/nu7042161 25825829 PMC4425138

[12] Gubbels JS, Kremers SP, van Kann DH, Stafleu A, Candel MJ, Dagnelie PC, et al Interaction between physical environment, social environment, and child characteristics in determining physical activity at child care. Health Psychol. 2011;30(1):84–90. 10.1037/a0021586 21133542

[13] Gubbels JS, Kremers SP, Stafleu A, Dagnelie PC, de Vries NK, Thijs C. Child-care environment and dietary intake of 2- and 3-year-old children. J Hum Nutr Diet. 2010;23(1):97–101. 10.1111/j.1365-277X.2009.01022.x 19943841

[14] Gubbels JS, Van Kann DH, de Vries NK, Thijs C, Kremers SP. The next step in health behavior research: the need for ecological moderation analyses—an application to diet and physical activity at childcare. Int J Behav Nutr Phys Act. 2014;11:52 10.1186/1479-5868-11-52 24742167 PMC4002539

[15] Friedman SL, Wachs TD. Measuring Environment Across the Life Span Emerging Methods and Concepts. Washington: American Psychological Association; 1999.

[16] Wachs TD. The nature of nurture. Newbury Park, CA: Sage; 1992.

[17] Kremers SP. Theory and practice in the study of influences on energy balance-related behaviors. Patient Educ Couns. 2010;79(3):291–8. 10.1016/j.pec.2010.03.002 20371159

[18] Bradley RH, Vandell DL. Child care and the well-being of children. Arch Pediatr Adolesc Med. 2007;161(7):669–76. 10.1001/archpedi.161.7.669 17606830

[19] Bronfenbrenner U. The Ecology of Human Development: Experiments by Nature and Design. Cambridge, MA: Harvard University Press; 1979.

[20] Tucker P, van Zandvoort MM, Burke SM, Irwin JD. The influence of parents and the home environment on preschoolers’ physical activity behaviours: a qualitative investigation of childcare providers’ perspectives. BMC Public Health. 2011;11:168 10.1186/1471-2458-11-168 21414218 PMC3070650

[21] Lloyd-Williams F, Bristow K, Capewell S, Mwatsama M. Young children’s food in Liverpool day-care settings: a qualitative study of pre-school nutrition policy and practice. Public Health Nutr. 2011;14(10):1858–66. 10.1017/S1368980011000619 21557874

[22] Fees B, Trost S, Bopp M, Dzewaltowski DA. Physical activity programming in family child care homes: providers’ perceptions of practices and barriers. J Nutr Educ Behav. 2009;41(4):268–73. 10.1016/j.jneb.2008.01.013 19508932

[23] Wilke S, Opdenakker C, Kremers SPJ, Gubbels JS. Factors influencing child care workers’ promotion of physical activity in children aged 0–4 years: a qualitative study. Early Years. 2013.

[24] O’Connor JP, Temple VA. Constraints and facilitators for physical activity in family day care. Australian Journal of Early Childhood. 2005;30(4):1–9.

[25] Määttä S, Ray C, Roos G, Roos E. Applying a Socioecological model to understand preschool children’s sedentary behaviors from the viewpoints of parents and preschool personnel. Early Childhood Educ J. 2015.

[26] Copeland KA, Sherman SN, Kendeigh CA, Kalkwarf HJ, Saelens BE. Societal Values and Policies May Curtail Preschool Children’s Physical Activity in Child Care Centers. Pediatrics. 2012;129(2):265–74. 10.1542/peds.2011-2102 22218842 PMC3269117

[27] Hesketh KR, Lakshman R, van Sluijs EMF. Barriers and facilitators to young children’s physical activity and sedentary behaviour: a systematic review and synthesis of qualitative literature. Obes Rev. 2017;18(9):987–1017. 10.1111/obr.12562 28589678 PMC5575514

[28] Gerritsen S, Wall C, Morton S. Child-care nutrition environments: results from a survey of policy and practice in New Zealand early childhood education services. Public Health Nutr. 2016;19(9):1531–42. 10.1017/S1368980015002955 26466671 PMC10271085

[29] Hirsch T, Lim C, Otten JJ. What’s for lunch? A socio-ecological approach to childcare nutrition. DIS '16 Proceedings of the 2016 ACM Conference on Designing Interactive Systems. 2016:1160–71.

[30] Dev DA, McBride BA, Speirs KE, Blitch KA, Williams NA. "Great Job Cleaning Your Plate Today!" Determinants of Child-Care Providers’ Use of Controlling Feeding Practices: An Exploratory Examination. Journal of the Academy of Nutrition and Dietetics. 2016;116(11):1803–9. 10.1016/j.jand.2016.07.016 27650534

[31] Johnson SL, Ramsay S, Armstrong Shultz J, Branen LJ, Fletcher JW. Creating potential for common ground and communication between early childhood prgram staff and parents about young children’s eating. J Nutr Educ Behav. 2013;45:558–70. 10.1016/j.jneb.2013.02.009 23769298

[32] Lumeng JC, Kaplan-Sanoff M, Shuman S, Kannan S. Head Start teachers’ perceptions of children’s eating behavior and weight status in the context of food scarcity. J Nutr Educ Behav. 2008;40:237–43. 10.1016/j.jneb.2007.07.001 18565464

[33] Baumgartner J, McBride B. Exploring parental philosophies regarding childcare: overlap orientations and the influence of childcare programmes on families. Early Child Development and Care. 2009;179(7).

[34] Mena NZ, Gorman K, Dickin K, Greene G, Tovar A. Contextual and cultural influences on parental feeding practices and involvement in child care centers among Hispanic parents. Childhood obesity. 2015;11(4):347–54. 10.1089/chi.2014.0118 25951503

[35] Nader PR, Huang TT, Gahagan S, Kumanyika S, Hammond RA, Christoffel KK. Next steps in obesity prevention: altering early life systems to support healthy parents, infants, and toddlers. Childhood obesity. 2012;8(3):195–204. 10.1089/chi.2012.0004 22799545

[36] Berge JM, Wall M, Bauer KW, Neumark-Sztainer D. Parenting characteristics in the home environment and adolescent overweight: a latent class analysis. Obesity (Silver Spring). 2010;18(4):818–25. 19816417 10.1038/oby.2009.324 PMC2893139

[37] Gevers DW, van Assema P, Sleddens EF, de Vries NK, Kremers SP. Associations between general parenting, restrictive snacking rules, and adolescent’s snack intake. The roles of fathers and mothers and interparental congruence. Appetite. 2015;87:184–91. 10.1016/j.appet.2014.12.220 25555538

[38] Sleddens EF, O’Connor TM, Watson KB, Hughes SO, Power TG, Thijs C, et al Development of the Comprehensive General Parenting Questionnaire for caregivers of 5–13 year olds. Int J Behav Nutr Phys Act. 2014;11:15 10.1186/1479-5868-11-15 24512450 PMC3926334

[39] Musher-Eizenman D, Holub S. Comprehensive Feeding Practices Questionnaire: validation of a new measure of parental feeding practices. J Pediatr Psychol. 2007;32(8):960–72. 10.1093/jpepsy/jsm037 17535817

[40] O’Connor TM, Cerin E, Hughes SO, Robles J, Thompson DI, Mendoza JA, et al Psychometrics of the preschooler physical activity parenting practices instrument among a Latino sample. Int J Behav Nutr Phys Act. 2014;11:3 10.1186/1479-5868-11-3 24428935 PMC3903032

[41] Gubbels JS, Sleddens EF, Raaijmakers L, Gies JM, Kremers SP. The Child-care Food and Activity Practices Questionnaire (CFAPQ): development and first validation steps. Public Health Nutr. 2016;19(11):1964–75. 10.1017/S1368980015003444 26634610 PMC4990721

[42] Portney LG, Watkins MP. Foundations of clinical research: Applications to practice (2nd edition). Upper Saddle River, NJ: Prentice Hall; 2000.

[43] Van Cauwenberghe E, Gubbels J, De Bourdeaudhuij I, Cardon G. Feasibility and validity of accelerometer measurements to assess physical activity in toddlers. Int J Behav Nutr Phys Act. 2011;8:67 10.1186/1479-5868-8-67 21703004 PMC3132156

[44] Trost SG, Fees BS, Haar SJ, Murray AD, Crowe LK. Identification and validity of accelerometer cut-points for toddlers. Obesity (Silver Spring). 2012;20(11):2317–9. 22173573 10.1038/oby.2011.364

[45] Pate RR, Almeida MJ, McIver KL, Pfeiffer KA, Dowda M. Validation and calibration of an accelerometer in preschool children. Obesity (Silver Spring). 2006;14(11):2000–6. 17135617 10.1038/oby.2006.234

[46] Fredriks AM, van Buuren S, Wit JM, Verloove-Vanhorick SP. Body index measurements in 1996–7 compared with 1980. Arch Dis Child. 2000;82(2):107–12. 10.1136/adc.82.2.107 10648362 PMC1718204

[47] Johnson SL, Birch LL. Parents’ and children’s adiposity and eating style. Pediatrics. 1994;94(5):653–61. 7936891

[48] Van IJzendoorn MH, Tavecchio LWC, Stams GJ, Verhoeven M, Reiling E. Attunement Between Parents and Professional Caregivers: A Comparison of Childrearing Attitudes in Different Child-care Settings. Journal of Marriage and the Family. 1998;60:771–81.

[49] Feagans LV, Manlove EE. Parents, infants, and day-care teachers: interrelations and implications for better child-care. Journal of Applied Developmental Psychology. 1994;15:585–602.

[50] Ghazvini AS, Readdick CA. Parent-Caregiver communication and quality of care in diverse child care settings. Early Childhood Research Quarterly. 1994;9:207–22.

[51] Shpancer N. The home-daycare link: Mapping children’s new world order. Early Childhood Research Quarterly. 2002;17:374–92.

[52] Dev DA, Byrd-Williams C, Ramsay S, McBride B, Srivastava D, Murriel A, et al Engaging Parents to Promote Children’s Nutrition and Health. Am J Health Promot. 2017;31(2):153–62. 10.1177/0890117116685426 28423928

[53] Gerritsen S, Morton SM, Wall CR. Physical activity and screen use policy and practices in childcare: results from a survey of early childhood education services in New Zealand. Aust N Z J Public Health. 2016;40(4):319–25. 10.1111/1753-6405.12529 27198060

[54] Benjamin Neelon SE, Mayhew M, O’Neill JR, Neelon B, Li F, Pate RR. Comparative Evaluation of a South Carolina Policy to Improve Nutrition in Child Care. Journal of the Academy of Nutrition and Dietetics. 2016;116(6):949–56. 10.1016/j.jand.2015.10.026 26777469

[55] Ward DS, Welker E, Choate A, Henderson KE, Lott M, Tovar A, et al Strength of obesity prevention interventions in early care and education settings: A systematic review. Prev Med. 2017;95 Suppl:S37–S52. 27693295 10.1016/j.ypmed.2016.09.033

[56] Ho M, Garnett SP, Baur L, Burrows T, Stewart L, Neve M, et al Effectiveness of lifestyle interventions in child obesity: systematic review with meta-analysis. Pediatrics. 2012;130(6):e1647–71. 10.1542/peds.2012-1176 23166346

[57] Okely AD, Hammersley ML. School-home partnerships: the missing piece in obesity prevention. Lancet Child Adolesc Health. 2017. 10.1016/S2352-4642(17)30154-2 30169195

[58] Votruba-Drzal E, Levine Coley R, Chase-Lansdale L. Child care and low-income children’s development: direct and moderated effects. Child Development. 2004;75(1):296–312. 15015691 10.1111/j.1467-8624.2004.00670.x

[59] Hawe P, Shiell A, Riley T. Theorising interventions as events in systems. Am J Community Psychol. 2009;43(3–4):267–76. 10.1007/s10464-009-9229-9 19390961

[60] Boonpleng W, Park CG, Gallo AM, Corte C, McCreary L, Bergren MD. Ecological influences of early childhood obesity: a multilevel analysis. West J Nurs Res. 2013;35(6):742–59. 10.1177/0193945913480275 23493675

[61] Davison KK, Jurkowski JM, Lawson HA. Reframing family-centred obesity prevention using the Family Ecological Model. Public Health Nutr. 2012:1–9. 23089267 10.1017/S1368980012004533 PMC5500251

[62] Hawkins SS, Cole TJ, Law C, Millennium Cohort Study Child Health G. An ecological systems approach to examining risk factors for early childhood overweight: findings from the UK Millennium Cohort Study. J Epidemiol Community Health. 2009;63(2):147–55. 10.1136/jech.2008.077917 18801795 PMC2678539

[63] Spence JC, Lee RE. Toward a comprehensive model of physical activity. Psychology of Sport and Exercise. 2003;4:7–24.

[64] Khandpur N, Charles J, Davison KK. Fathers’ Perspectives on Coparenting in the Context of Child Feeding. Childhood obesity. 2016;12(6):455–62. 10.1089/chi.2016.0118 27636332 PMC6445205

[65] De Schipper J, Tavecchio LWC, Van IJzendoorn MH, Van Zeijl J. Goodness-of-fit in center day care: relations of temperament, stability, and quality of care with the child’s adjustment. Early Childhood Research Quarterly. 2004;19:257–72.

[66] Rollins BY, Loken E, Savage JS, Birch LL. Effects of restriction on children’s intake differ by child temperament, food reinforcement, and parent’s chronic use of restriction. Appetite. 2014;73:31–9. 10.1016/j.appet.2013.10.005 24511616 PMC4578816

[67] Gubbels JS, Kremers SP, Stafleu A, Dagnelie PC, Goldbohm RA, de Vries NK, et al Diet-related restrictive parenting practices. Impact on dietary intake of 2-year-old children and interactions with child characteristics. Appetite. 2009;52(2):423–9. 10.1016/j.appet.2008.12.002 19114065

