# Supplementary material for: Energy balance-related parenting and child-care practices: The importance of meso-system consistency
Source: PLoS One. 2018 Sep 7;13(9):e0203689. doi: 10.1371/journal.pone.0203689 (PMC6128647; doi:10.1371/journal.pone.0203689)
Supplement: S1 File — (DOC) [file pone.0203689.s001.doc]

**Supporting information 1: CFPQ, PPAPP and CFAPQ items for parents and childcare staff in Dutch and English**

**Parental questions in Dutch**

**De volgende vragen gaan over hoe u omgaat met bewegen van uw kind. *(houdt bij het invullen uw oudste kind dat naar het kinderdagverblijf gaat in gedachten).***

| **Hoe vaak..** | **Nooit** | **Zelden** | **Soms** | **Vaak** | **Altijd** |
| --- | --- | --- | --- | --- | --- |
| 1. .. geeft u een voorbeeld voor uw kind door zelf in het bijzijn van uw kind lichamelijk actief te zijn? | O | O | O | O | O |
| 2. .. speelt u actieve spelletjes met uw kind? *(bijv. een balspel of rennen)* | O | O | O | O | O |
| 3. .. laat u uw kind niet actief spelen omdat u niet wilt dat hij/zij vies wordt? | O | O | O | O | O |
| 4. .. zegt u tegen uw kind dat hij/zij een sport of beweegspelletje (nog) niet kan of er (nog) niet goed genoeg in is? | O | O | O | O | O |
| 5. .. doet u leuke beweegspelletjes om uw kind actief bezig te laten zijn? | O | O | O | O | O |
| 6. .. zegt u positieve dingen om uw kind te motiveren om te bewegen? | O | O | O | O | O |
| 7. .. leert u uw kind nieuwe manieren om te bewegen? | O | O | O | O | O |
| 8. .. speelt u samen als gezin een sport of beweegspelletje? | O | O | O | O | O |
| 9. .. geeft u uw kind keuzes welke lichamelijke activiteit hij/zij wil doen? | O | O | O | O | O |
| 10. .. plant u tijd in voor actieve speeltijd? | O | O | O | O | O |
| 11. .. laat u uw kind een beweegspelletje kiezen om samen te doen? | O | O | O | O | O |
| 12. .. danst u met uw kind? | O | O | O | O | O |
| 13. .. vertelt u uw kind dat hij/zij zich kan bezeren als hij/zij actief speelt? | O | O | O | O | O |
| 14. .. doet u een beweegspelletje met uw kind? *(bijv. voetbal)* | O | O | O | O | O |
| 15. .. corrigeert u uw kind omdat hij/zij te bewegelijk/druk is? | O | O | O | O | O |
| 16. ..leert u uw kind dat bewegen goed is voor zijn/haar gezondheid? | O | O | O | O | O |
| 17. ..beloont u uw kind als hij/zij zich rustig houdt? | O | O | O | O | O |
| 18. ..heeft u buitenspeelgoed beschikbaar voor uw kind *(bijv. springtouw, voetbal)*? | O | O | O | O | O |
| . ..zet u uw kind in een buggy i.p.v. hem/haar te laten lopen of kruipen? | O | O | O | O | O |
| 20. ..draagt u uw kind omdat hij/zij niet wil lopen of kruipen? | O | O | O | O | O |

***De volgende vragen gaan over hoe u omgaat met de voeding van uw kind. Er zijn geen goede of foute antwoorden, kies het antwoord dat het meest van toepassing is.*** *(houdt bij het invullen uw oudste kind dat naar het kinderdagverblijf gaat in gedachten).*

| **Hoe vaak…** | | **Nooit** | | **Zelden** | | **Soms** | | **Vaak** | **Altijd** |
| --- | --- | --- | --- | --- | --- | --- | --- | --- | --- |
| 1. ...laat u uw kind eten wat hij/zij wil? | | O | | O | | O | | O | O |
| 2. ..laat u tijdens de maaltijden uw kind kiezen wat hij/zij wil uit het eten dat wordt geserveerd? *(bijv. zelf het beleg kiezen tijdens de lunch)* | | O | | O | | O | | O | O |
| 3. ..geeft u uw kind snel iets te eten of te drinken als hij/zij vervelend is? | | O | | O | | O | | O | O |
| 4. ..laat u uw kind van tafel gaan als hij/zij vol zit, zelfs als de rest van het gezin niet klaar is met eten? | | O | | O | | O | | O | O |
| 5. ..geeft u uw kind iets te eten of drinken als hij/zij van streek is, zelfs als u denkt dat hij/zij geen honger heeft? | | O | | O | | O | | O | O |
| 6. ..maakt u iets anders klaar als uw kind het eten wat u geeft niet lekker vindt? | | O | | O | | O | | O | O |
| 7. ..laat u uw kind tussendoortjes eten wanneer hij/zij dat wil? | | O | | O | | O | | O | O |
| 8. ..geeft u uw kind iets te eten of drinken als hij/zij zich verveelt, zelfs als u denkt dat hij/zij geen honger heeft? | | O | | O | | O | | O | O |
| 9. ..moedigt u uw kind aan om eerst gezonde producten te eten en daarna pas ongezonde producten? *(bijv. eerst een boterham met hartig beleg, daarna pas met zoet beleg).* | | O | | O | | O | | O | O |
|  | |  | |  | |  | |  |  |
| **2. Hierna volgen een aantal stellingen over hoe u omgaat met de voeding van uw kind. Geef aan in hoeverre u het eens bent met de stellingen. Er zijn geen goede of foute antwoorden.** (*houdt bij het invullen uw oudste kind dat naar het kinderdagverblijf gaat in gedachten).* | | | | | | | | | |
|  | **Hele-maal mee oneens** | | **Een beetje mee oneens** | | **Neu-traal** | | **Een beetje mee eens** | | **Hele-maal mee eens** |
| 1. De meeste voedingsproducten die ik in huis heb zijn gezond. | O | | O | | O | | O | | O |
| 2. Ik heb veel snacks in huis *(bijv. crackers, chips, kaasstengels).* | O | | O | | O | | O | | O |
| 3. Mijn kind moet altijd zijn/haar bord helemaal leeg eten. | O | | O | | O | | O | | O |
| 4. Ik wil er zeker van zijn dat mijn kind niet teveel vette producten eet. *(bijv. kaas, worst, koek)* | O | | O | | O | | O | | O |
| 5. Ik laat mijn kind zien dat ik van gezond eten houd. | O | | O | | O | | O | | O |
| 6.Bij iedere maaltijd die thuis wordt gegeten, kan mijn kind kiezen uit veel verschillende soorten gezonde voedingsproducten | O | | O | | O | | O | | O |
| 7. Ik moedig mijn kind aan om nieuwe voedingsproducten te proberen. | O | | O | | O | | O | | O |
| 8. Ik bespreek met mijn kind waarom het belangrijk is om gezond te eten | O | | O | | O | | O | | O |
| 9. Ik vertel mijn kind dat gezond eten lekker is. | O | | O | | O | | O | | O |
| 10. Als mijn kind zegt: “Ik heb geen honger”, probeer ik hem/haar toch te laten eten. | O | | O | | O | | O | | O |
| 11. Ik bespreek de voedingswaarde van voedingsproducten met mijn kind *(bijv. “in hagelslag zit veel suiker”, of “in fruit zitten veel vitamines”)* | O | | O | | O | | O | | O |
| 12. Als mijn kind meer eet dan normaal tijdens een eetmoment, probeer ik zijn/haar voedingsinname te beperken bij het volgende eetmoment. | O | | O | | O | | O | | O |
| 13. Ik heb veel zoetigheid in huis *(bijv. koek, snoep, ijs).* | O | | O | | O | | O | | O |
| 14. Ik moedig mijn kind aan om veel verschillende soorten voedingsproducten te eten. | O | | O | | O | | O | | O |
| 15. Ik probeer gezond te eten in het bijzijn van mijn kind, zelfs als ik het zelf niet zo lekker vind. | O | | O | | O | | O | | O |
| 16. Ik wil er zeker van zijn dat mijn kind niet teveel van zijn/haar favoriete producten eet. | O | | O | | O | | O | | O |
| 17. Ik vertel mijn kind wat hij/zij wel en niet moet eten, zonder uitleg. | O | | O | | O | | O | | O |
| 18. Ik wil er zeker van zijn mijn kind niet teveel zoetigheid eet. *(bijv. koek, snoep, ijs)* | O | | O | | O | | O | | O |
| 19. Ik zorg ervoor dat ik het goede voorbeeld ben voor mijn kind door zelf gezond te eten. | O | | O | | O | | O | | O |
| 20. Als mijn kind maar een kleine portie eet, probeer ik hem/haar meer te laten eten. | O | | O | | O | | O | | O |
| 21. Ik probeer enthousiast te zijn over het eten van gezonde voeding. | O | | O | | O | | O | | O |
| 22. Als ik het eetgedrag van mijn kind niet zou sturen, zou hij/zij teveel van zijn/haar favoriete producten eten. | O | | O | | O | | O | | O |
| 23. Als mijn kind zegt dat hij/zij klaar is met eten, probeer ik hem/haar nog een hapje te laten eten. | O | | O | | O | | O | | O |

**Parental questions in English**

**The next questions are about how you handle physical activity of your child. *(keep your oldest child that uses child-care in mind while filling out the questions).***

| **How often…** | **Never** | **Rarely** | **Sometimes** | **Mostly** | **Always** |
| --- | --- | --- | --- | --- | --- |
| 1. .. do you set an example for your child by exercising in front of him/her? | O | O | O | O | O |
| 2. .. do you play active games with your child *(such as playing ball or racing)* | O | O | O | O | O |
| 3. .. do you not let your child play actively for fear of him/her getting dirty? | O | O | O | O | O |
| 4. .. do you tell your child he/she is not good enough (yet) at sports or active games? | O | O | O | O | O |
| 5. .. do you find appropriate games that get your child moving? | O | O | O | O | O |
| 6. .. do you say positive things to motivate your child to be more active? | O | O | O | O | O |
| 7. .. do you teach your child new ways to be active? | O | O | O | O | O |
| 8. .. do you play a sport or active game together as a family? | O | O | O | O | O |
| 9. .. do you give your child choices of what physical activities to do? | O | O | O | O | O |
| 10. .. do you set time aside for active play? | O | O | O | O | O |
| 11. .. do you allow your child to pick an active game to do together? | O | O | O | O | O |
| 12. .. do you dance with your child? | O | O | O | O | O |
| 13. .. do you tell your child he/she will get hurt if he/she plays actively? | O | O | O | O | O |
| 14. .. do you play sports games with your child? *(such as soccer)* | O | O | O | O | O |
| 15. .. do you discipline your child for being too active? | O | O | O | O | O |
| 16. ..do you teach your child that being active is good for his/her health? | O | O | O | O | O |
| 17. ..do you reward your child for being still | O | O | O | O | O |
| 18. ..do you have outdoor toys available for your child *(such as jumping rope, ball)*? | O | O | O | O | O |
| 19. ..do you put your child in a stroller instead of letting him/her walk or crawl? | O | O | O | O | O |
| 20. ..do you carry your child because he/she does not want to walk or crawl? | O | O | O | O | O |

**The next questions are about how you handle dietary intake of your child. There are no right or wrong answers, choose the answer that applies most.** *(keep your oldest child that uses child-care in mind while filling out the questions).*

| **How often…** | | **Never** | | **Rarely** | | **Sometimes** | | **Mostly** | **Always** |
| --- | --- | --- | --- | --- | --- | --- | --- | --- | --- |
| 1. ...do you let your child eat whatever he/she wants? | | O | | O | | O | | O | O |
| 2. ..at meals, do you let your child choose the foods he/she wants from what is served? *(such as choosing the bread toppings during lunch)* | | O | | O | | O | | O | O |
| 3. .. when this child gets fussy, is giving him/her something to eat the first thing you do? | | O | | O | | O | | O | O |
| 4. ..do you allow your child to leave the table when he/she is full, even if your family is not done eating? | | O | | O | | O | | O | O |
| 5. .. do you give your child something to eat or drink if he/she is upset even if you think he/she is not hungry | | O | | O | | O | | O | O |
| 6. ..do you make something else if your child does not like what is being served? | | O | | O | | O | | O | O |
| 7. ..do you allow your child to eat snacks whenever he/she wants? | | O | | O | | O | | O | O |
| 8. .. do you give this child something to eat or drink if he/she is bored even if you think he/she is not hungry ? | | O | | O | | O | | O | O |
| 9. ..do you encourage your child to eat healthy foods before unhealthy ones? *(such as first eathing a sandwich with a savory topping before eating a sweet topping).* | | O | | O | | O | | O | O |
|  | |  | |  | |  | |  |  |
| **Following are statements about how you handle your child’s dietary intake. Indicate to what extent you agree with the statements. There are no right or wrong answers.** *(keep your oldest child that uses child-care in mind while filling out the questions).* | | | | | | | | | |
|  | **Disagree** | | **Slightly disagree** | | **Neutral** | | **Slightly agree** | | **Agree** |
| 1. Most of the food I keep in the house is healthy | O | | O | | O | | O | | O |
| 2. I keep a lot of snack food in my house *(such as crackers, potato chips, cheese strings).* | O | | O | | O | | O | | O |
| 3. My child should always eat all of the food on his/her plate. | O | | O | | O | | O | | O |
| 4. I have to be sure that my child does not eat too many high-fat foods. *(such as cheese, sausage, cake)* | O | | O | | O | | O | | O |
| 5. I show my child how much I enjoy eating healthy foods. | O | | O | | O | | O | | O |
| 6. A variety of healthy foods are available to my child at each meal served at home. | O | | O | | O | | O | | O |
| 7. I encourage my child to try new foods. | O | | O | | O | | O | | O |
| 8. I discuss with my child why it is important to eat healthily. | O | | O | | O | | O | | O |
| 9. I tell my child that healthy food tastes good. | O | | O | | O | | O | | O |
| 10. If my child says ‘I’m not hungry’, I try to get him/her to eat anayway. | O | | O | | O | | O | | O |
| 11. I discuss the nutritional content of food products with my child *(such as ‘Sprinkles have a lot of sugar’ or ‘fruit contains vitamines’)* | O | | O | | O | | O | | O |
| 12. If my child eats more than usual at one meal, I try to restrict his/her eating at the next meal. | O | | O | | O | | O | | O |
| 13. I keep a lot of sweets in my house *(cookies, sweets, ice cream)*. | O | | O | | O | | O | | O |
| 14. I encourage my child to eat a variety of foods. | O | | O | | O | | O | | O |
| 15. I try to eat healthy foods in front of my child, even if they are not my favorite. | O | | O | | O | | O | | O |
| 16. I have to be sure that my child does not eat too much of his/her favorite foods. | O | | O | | O | | O | | O |
| 17. I tell my child what to eat and what not to eat without explanation. | O | | O | | O | | O | | O |
| 18. I have to be sure that my child does not eat too many sweets *(such as candy, cookies, ice cream)* | O | | O | | O | | O | | O |
| 19. I model healthy eating for my child by eating healthy foods myself. | O | | O | | O | | O | | O |
| 20. If my child eats only a small helping, I try to get him/her to eat more. | O | | O | | O | | O | | O |
| 21. I try to show enthousiasm about eating healthy foods. | O | | O | | O | | O | | O |
| 22. If I did not guide or regulate my child’s eating, he/she would eat too much of his/her favorite foods. | O | | O | | O | | O | | O |
| 23. When he/she says he/she is finished eating, I try to get my child to eat one more bite of food. | O | | O | | O | | O | | O |

**Child-care staff questions in Dutch**

**De volgende vragen gaan over hoe u omgaat met bewegen van de kinderen. Probeer bij deze vragen het antwoord te geven dat voor de meeste kinderen van toepassing is.**

| **Hoe vaak..** | **Nooit** | **Zelden** | **Soms** | **Vaak** | **Altijd** |
| --- | --- | --- | --- | --- | --- |
| 1. .. geeft u een voorbeeld voor de kinderen door zelf in het bijzijn van de kinderen lichamelijk actief te zijn? | O | O | O | O | O |
| 2. .. speelt u actieve spelletjes met de kinderen? *(bijv. een balspel of rennen)* | O | O | O | O | O |
| 3. .. laat u kinderen niet actief spelen omdat u niet wilt dat ze vies worden? | O | O | O | O | O |
| 4. .. zegt u tegen kinderen dat ze een sport of beweegspelletje (nog) niet kunnen of er (nog) niet goed genoeg in zijn? | O | O | O | O | O |
| 5. .. doet u leuke beweegspelletjes om de kinderen actief bezig te laten zijn? | O | O | O | O | O |
| 6. .. zegt u positieve dingen om kinderen te motiveren om te bewegen? | O | O | O | O | O |
| 7. .. leert u de kinderen nieuwe manieren om te bewegen? | O | O | O | O | O |
| 8. .. speelt u samen met de kinderen (en eventueel andere pedagogisch medewerkers) een sport of beweegspelletje? | O | O | O | O | O |
| 9. .. geeft u de kinderen keuzes welke lichamelijke activiteit ze willen doen? | O | O | O | O | O |
| 10. .. plant u tijd in voor actieve speeltijd? | O | O | O | O | O |
| 11. .. laat u kinderen een beweegspelletje kiezen om samen te doen? | O | O | O | O | O |
| 12. .. danst u met de kinderen? | O | O | O | O | O |
| 13. .. vertelt u kinderen dat ze zich kunnen bezeren als ze actief speelt? | O | O | O | O | O |
| 14. .. doet u een beweegspelletje met de kinderen? *(bijv. voetbal)* | O | O | O | O | O |
| 15. .. corrigeert u kinderen omdat ze te bewegelijk/druk zijn? | O | O | O | O | O |
| 16. ..leert u de kinderen dat bewegen goed is voor hun gezondheid? | O | O | O | O | O |
| 17. ..beloont u kinderen als ze zich rustig houden? | O | O | O | O | O |
| 18. ..heeft u buitenspeelgoed beschikbaar voor de kinderen *(bijv. springtouw, voetbal)*? | O | O | O | O | O |
| 19. ..zet u kinderen in een buggy i.p.v. ze te laten lopen of kruipen? | O | O | O | O | O |
| 20. ..draagt u kinderen omdat ze niet willen lopen of kruipen? | O | O | O | O | O |

***De volgende vragen gaan over hoe u omgaat met de voeding van de kinderen. Probeer bij deze vragen het antwoord te geen dat voor de meeste kinderen van toepassing is. Er zijn geen goede of foute antwoorden.***

| **Hoe vaak…** | | **Nooit** | | **Zelden** | | **Soms** | | **Vaak** | **Altijd** |
| --- | --- | --- | --- | --- | --- | --- | --- | --- | --- |
| 1. ..laat u de kinderen eten wat ze willen? | | O | | O | | O | | O | O |
| 2. ..laat u tijdens de maaltijden de kinderen kiezen wat ze willen uit het eten dat wordt geserveerd? *(bijv. zelf het beleg kiezen tijdens de lunch)* | | O | | O | | O | | O | O |
| 3. ..geeft u kinderen snel iets te eten of te drinken als ze vervelend zijn? | | O | | O | | O | | O | O |
| 4. ..laat u kinderen van tafel gaan als ze vol zitten, zelfs als de rest niet klaar is met eten? | | O | | O | | O | | O | O |
| 5. ..geeft u kinderen iets te eten of drinken als ze van streek zijn, zelfs als u denkt dat ze geen honger hebben? | | O | | O | | O | | O | O |
| 6. ..maakt u iets anders klaar als kinderen het eten wat u geeft niet lekker vinden? | | O | | O | | O | | O | O |
| 7. ..laat u kinderen tussendoortjes eten wanneer zij dat willen? | | O | | O | | O | | O | O |
| 8. ..geeft u kinderen iets te eten of drinken als ze zich vervelen, zelfs als u denkt dat ze geen honger hebben? | | O | | O | | O | | O | O |
| 9. ..moedigt u de kinderen aan om eerst gezonde producten te eten en daarna pas ongezonde producten? *(bijv. eerst een boterham met hartig beleg, daarna pas met zoet beleg).* | | O | | O | | O | | O | O |
|  | |  | |  | |  | |  |  |
| **2. Hierna volgen een aantal stellingen over hoe u omgaat met de voeding van de kinderen. Geef aan in hoeverre u het eens bent met de stellingen. Probeer bij deze vragen het antwoord te geven dat voor de meeste kinderen van toepassing is. Er zijn geen goede of foute antwoorden. Het gaat om uw mening.** | | | | | | | | | |
|  | **Hele-maal mee oneens** | | **Een beetje mee oneens** | | **Neu-traal** | | **Een beetje mee eens** | | **Hele-maal mee eens** |
| 1. De meeste voedingsproducten op het kinderdagverblijf zijn gezond. | O | | O | | O | | O | | O |
| 2. Er zijn veel snacks aanwezig op het kinderdagverblijf *(bijv. crackers, chips, kaasstengels).* | O | | O | | O | | O | | O |
| 3. De kinderen moeten altijd hun bord helemaal leeg eten. | O | | O | | O | | O | | O |
| 4. Ik wil er zeker van zijn dat de kinderen niet teveel vette producten eten. *(bijv. kaas, worst, koek)* | O | | O | | O | | O | | O |
| 5. Ik laat de kinderen zien dat ik van gezond eten houd. | O | | O | | O | | O | | O |
| 6.Bij iedere maaltijd die op het kinderdagverblijf wordt gegeten, kunnen de kinderen kiezen uit veel verschillende soorten gezonde voedingsproducten | O | | O | | O | | O | | O |
| 7. Ik moedig de kinderen aan om nieuwe voedingsproducten te proberen. | O | | O | | O | | O | | O |
| 8. Ik bespreek de kinderen waarom het belangrijk is om gezond te eten | O | | O | | O | | O | | O |
| 9. Ik vertel de kinderen dat gezond eten lekker is. | O | | O | | O | | O | | O |
| 10. Als een kind zegt: “Ik heb geen honger”, probeer ik hem/haar toch te laten eten. | O | | O | | O | | O | | O |
| 11. Ik bespreek de voedingswaarde van voedingsproducten met de kinderen *(bijv. “in hagelslag zit veel suiker”, of “in fruit zitten veel vitamines”)* | O | | O | | O | | O | | O |
| 12. Als een kind meer eet dan normaal tijdens een eetmoment, probeer ik zijn/haar voedingsinname te beperken bij het volgende eetmoment. | O | | O | | O | | O | | O |
| 13. Er is veel zoetigheid aanwezig op het kinderdagverblijf *(bijv. koek, snoep, ijs).* | O | | O | | O | | O | | O |
| 14. Ik moedig de kinderen aan om veel verschillende soorten voedingsproducten te eten. | O | | O | | O | | O | | O |
| 15. Ik probeer gezond te eten in het bijzijn van de kinderen, zelfs als ik het zelf niet zo lekker vind. | O | | O | | O | | O | | O |
| 16. Ik wil er zeker van zijn dat de kinderen niet teveel van hun favoriete producten eten. | O | | O | | O | | O | | O |
| 17. Ik vertel de kinderen wat ze wel en niet moeten eten, zonder uitleg. | O | | O | | O | | O | | O |
| 18. Ik wil er zeker van zijn dat de kinderen niet teveel zoetigheid eten. *(bijv. koek, snoep, ijs)* | O | | O | | O | | O | | O |
| 19. Ik zorg ervoor dat ik het goede voorbeeld ben voor de kinderen ben door zelf gezond te eten. | O | | O | | O | | O | | O |
| 20. Als een kind maar een kleine portie eet, probeer ik hem/haar meer te laten eten. | O | | O | | O | | O | | O |
| 21. Ik probeer enthousiast te zijn over het eten van gezonde voeding. | O | | O | | O | | O | | O |
| 22. Als ik het eetgedrag van de kinderen niet zou sturen, zouden ze teveel van hun favoriete producten eten. | O | | O | | O | | O | | O |
| 23. Als een kind zegt dat hij/zij klaar is met eten, probeer ik hem/haar nog een hapje te laten eten. | O | | O | | O | | O | | O |

**Child-care staff questions in English**

**The next questions are about how you handle physical activity of the children. Try to give the answer that applies to the most children.**

| **How often…** | **Never** | **Rarely** | **Sometimes** | **Mostly** | **Always** |
| --- | --- | --- | --- | --- | --- |
| 1. .. do you set an example for the children by exercising in front of them? | O | O | O | O | O |
| 2. .. do you play active games with the children *(such as playing ball or racing)* | O | O | O | O | O |
| 3. .. do you not let children play actively for fear of them getting dirty? | O | O | O | O | O |
| 4. .. do you tell a child he/she is not good enough (yet) at sports or active games? | O | O | O | O | O |
| 5. .. do you find appropriate games that get the children moving? | O | O | O | O | O |
| 6. .. do you say positive things to motivate the children to be more active? | O | O | O | O | O |
| 7. .. do you teach the children new ways to be active? | O | O | O | O | O |
| 8. .. do you play a sport or active game together with the children (and perhaps other child-care staff)? | O | O | O | O | O |
| 9. .. do you give children choices of what physical activities to do? | O | O | O | O | O |
| 10. .. do you set time aside for active play? | O | O | O | O | O |
| 11. .. do you allow children to pick an active game to do together? | O | O | O | O | O |
| 12. .. do you dance with the children? | O | O | O | O | O |
| 13. .. do you tell a child he/she will get hurt if he/she plays actively? | O | O | O | O | O |
| 14. .. do you play sports games with the children? *(such as soccer)* | O | O | O | O | O |
| 15. .. do you discipline children for being too active? | O | O | O | O | O |
| 16. ..do you teach the children that being active is good for his/her health? | O | O | O | O | O |
| 17. ..do you reward children for being still | O | O | O | O | O |
| 18. ..do you have outdoor toys available for the children *(such as jumping rope, ball)*? | O | O | O | O | O |
| 19. ..do you put a child in a stroller instead of letting him/her walk or crawl? | O | O | O | O | O |
| 20. ..do you carry a child because he/she does not want to walk or crawl? | O | O | O | O | O |

***The next questions are about how you handle dietary intake of the children. Try to give the answer that applies to the most children. There are no right or wrong answers, choose the answer that applies most.***

| **How often…** | | **Never** | | **Rarely** | | **Sometimes** | | **Mostly** | **Always** |
| --- | --- | --- | --- | --- | --- | --- | --- | --- | --- |
| 1. ...do you let the children eat whatever they want? | | O | | O | | O | | O | O |
| 2. ..at meals, do you let children choose the foods they want from what is served? *(such as choosing the bread toppings during lunch)* | | O | | O | | O | | O | O |
| 3. .. when a child gets fussy, is giving him/her something to eat the first thing you do? | | O | | O | | O | | O | O |
| 4. ..do you allow children to leave the table when they are full, even if the rest is not done eating? | | O | | O | | O | | O | O |
| 5. .. do you give a child something to eat or drink if he/she is upset even if you think he/she is not hungry | | O | | O | | O | | O | O |
| 6. ..do you make something else if children do not like what is being served? | | O | | O | | O | | O | O |
| 7. ..do you allow children to eat snacks whenever they want? | | O | | O | | O | | O | O |
| 8. .. do you give a child something to eat or drink if he/she is bored even if you think he/she is not hungry ? | | O | | O | | O | | O | O |
| 9. ..do you encourage the children to eat healthy foods before unhealthy ones? *(such as first eathing a sandwich with a savory topping before eating a sweet topping).* | | O | | O | | O | | O | O |
|  | |  | |  | |  | |  |  |
| **Following are statements about how you handle the children’s dietary intake. Indicate to what extent you agree with the statements. There are no right or wrong answers. We are interested in your opinion.** | | | | | | | | | |
|  | **Disagree** | | **Slightly disagree** | | **Neutral** | | **Slightly agree** | | **Agree** |
| 1. Most of the food at the child-care center is healthy | O | | O | | O | | O | | O |
| 2. There is a lot of snack food present at the child-care center *(such as crackers, potato chips, cheese strings).* | O | | O | | O | | O | | O |
| 3. The children should always eat all of the food on their plate. | O | | O | | O | | O | | O |
| 4. I have to be sure that the children do not eat too many high-fat foods. *(such as cheese, sausage, cake)* | O | | O | | O | | O | | O |
| 5. I show the children how much I enjoy eating healthy foods. | O | | O | | O | | O | | O |
| 6. A variety of healthy foods are available to the children at each meal served at the child-care center. | O | | O | | O | | O | | O |
| 7. I encourage the children to try new foods. | O | | O | | O | | O | | O |
| 8. I discuss with the children why it is important to eat healthily. | O | | O | | O | | O | | O |
| 9. I tell the children that healthy food tastes good. | O | | O | | O | | O | | O |
| 10. If a child says ‘I’m not hungry’, I try to get him/her to eat anayway. | O | | O | | O | | O | | O |
| 11. I discuss the nutritional content of food products with the children *(such as ‘Sprinkles have a lot of sugar’ or ‘fruit contains vitamines’)* | O | | O | | O | | O | | O |
| 12. If a child eats more than usual at one meal, I try to restrict his/her eating at the next meal. | O | | O | | O | | O | | O |
| 13. There are a lot of sweets at the child-care center *(cookies, sweets, ice cream)*. | O | | O | | O | | O | | O |
| 14. I encourage the children to eat a variety of foods. | O | | O | | O | | O | | O |
| 15. I try to eat healthy foods in front of the children, even if they are not my favorite. | O | | O | | O | | O | | O |
| 16. I have to be sure that the children do not eat too much of their favorite foods. | O | | O | | O | | O | | O |
| 17. I tell the children what to eat and what not to eat without explanation. | O | | O | | O | | O | | O |
| 18. I have to be sure that the children do not eat too many sweets *(such as candy, cookies, ice cream)* | O | | O | | O | | O | | O |
| 19. I model healthy eating for the children by eating healthy foods myself. | O | | O | | O | | O | | O |
| 20. If a child eats only a small helping, I try to get him/her to eat more. | O | | O | | O | | O | | O |
| 21. I try to show enthousiasm about eating healthy foods. | O | | O | | O | | O | | O |
| 22. If I did not guide or regulate the children’s eating, they would eat too much of their favorite foods. | O | | O | | O | | O | | O |
| 23. When they says they are finished eating, I try to get the children to eat one more bite of food. | O | | O | | O | | O | | O |
